# *Babesia canis* spp. in dogs in Baghdad Province, Iraq: First molecular identification and clinical and epidemiological study

**DOI:** 10.14202/vetworld.2020.579-585

**Published:** 2020-03-28

**Authors:** Naseir Mohammed Badawi, Afaf Abdulrahman Yousif

**Affiliations:** Department of Internal and Preventive Veterinary Medicine, College of Veterinary Medicine, University of Baghdad, Baghdad, Iraq

**Keywords:** 18S rRNA gene, *Babesia canis*, Baghdad, dog, Iraq

## Abstract

**Aims:**

The aim of this study was to investigate babesiosis in dogs of different breeds and ages and of both sexes in Baghdad Province by molecular detection of *Babesia canis* using conventional polymerase chain reaction (PCR) and sequencing followed by phylogenetic analyses.

**Materials and Methods:**

Blood samples were collected from 310 dogs of different ages and breeds, and of both sexes in different areas of Baghdad Province from December 2018 to September 2019; during clinical examinations, body temperature, pulse, respiratory rate, and signs of diseases were recorded. PCR was used to amplify a specific 450-bp fragment of the 18S rRNA gene of *B. canis*. PCR products were sequenced, and MEGA 6.0 software was used for analysis. Chi-square and odds ratio tests were used to investigate the prevalence and risk factors of babesiosis.

**Results:**

Clinical signs of babesiosis included paleness or icterus of the mucus membranes, tick infestation, and febrile illness during the acute and subacute phase. The prevalence of infection with *B. canis* was 5.1%, with the higher prevalence in male dogs and in dogs <3 years of age. Huskies were more likely to be infected than other dogs. Infection prevalence was highest in April and June and was higher in spring and summer than in winter. Using sequence data, 14 isolates of *Babesia canis canis* and one isolate of each *Babesia canis rossi* and *Babesia canis vogeli* were identified. Phylogenetic analyses of *B. canis* *canis* revealed that three shared clades and several isolated lineages were similar to other isolates (97-99% similarity), whereas *B. canis vogeli* and *B. canis rossi* showed similarities of 98% and 99% with isolates from other geographical regions.

**Conclusion:**

This study provides the first molecular record and phylogenic analysis of *B. canis* in dogs in Iraq, and it will be valuable for confirming clinical signs and studying epidemiological risk factors of babesiosis in dogs.

## Introduction

Canine piroplasms belong to two distinct species – the large *Babesia canis*, which measures 4-5 μm and is typically pear-shaped, and the small round *Babesia gibsoni* measuring 1-2.5 μm. *B. canis*, according to antigenic properties, is sub-divided into three subspecies – *Babesia canis canis* which occurs in Europe, *Babesia canis vogeli* occurring in tropical and subtropical regions, and *Babesia canis rossi* which is found in South Africa [1]. *Babesia* can be transmitted by ticks, dog bites, and blood transfusions, and the pathogen can transgress the placental barrier [2]. Vertical transmission of *B. canis* (i.e., congenital infection) was shown by Mierzejewska *et al*. [3], who detected *B. canis* in pups of infected mother 7 weeks postpartum, and isolates from pups were 100% homologous with those of their mothers. Vectors transmitting *B. canis* include *Rhipicephalus sanguineus*, *Dermacentor reticulatus*, and *Haemaphysalis elliptica*; small *Babesia* species in dogs, such as *B. gibsoni, Babesia conradae*, and *Babesia microti*-like species, can be transmitted by *Haemaphysalis bispinosa, Haemaphysalis longicornis*, wildlife reservoirs, and *Ixodes hexagonus* [4].

The first molecular study on *B. canis* in Iran was conducted by Bigdeli *et al*. [5], who found only one out of 280 dogs to be infected. In Turkey, *Babesia* was first detected in two dogs, according to clinical signs and blood smears, which was subsequently confirmed by polymerase chain reaction (PCR) [6]. The classic method of detecting *B. canis* using blood smears is considered the gold standard in acute and subacute cases [7]. Positive cases, as detected by blood smear, are necessarily also positive by PCR detection, whereas false-negative results obtained by microscopy may be found to be positive using PCR tests [8]. The specific primers PIRO-A1 and PIRO-B have been used previously to amplify a 450-bp region of the 18S rRNA gene in *B. canis* and other *Babesia* species [9-15].

So far, no molecular studies on babesiosis in dogs have been conducted in Iraq, and all the previous studies on this disease were conducted using blood smear diagnosis. The first study reporting *Piroplasma canis* in a blood smear of one dog in Mesopotamia (present Iraq) was conducted in 1920 [16], and the first descriptions of *B*. *gibsoni* and *B*. *canis* in blood smears of dogs were produced in Nineveh (Mosul), Iraq [17]. Infection with *B. gibsoni* in Baghdad, Iraq, occurred in 52 out of 108 dogs, as diagnosed by Al-Taie and Fadhil [18] using blood smears.

In the present study, we investigated clinical and epidemiological parameters of babesiosis in dogs and, for the first time, performed molecular detection, sequencing, and phylogenetic analyses of *B. canis* in Iraq.

## Materials and Methods

### Ethical approval

All procedures used in the present study were approved by the Committee of College of Veterinary Medicine, University of Baghdad, approval number 7/2018.

### Animals and clinical examination

Three hundred and ten dogs were examined at Baghdad Veterinary Hospital from December 2018 to September 2019. The dogs included 193 males and 117 females; 171 dogs were younger than 3 years and 139 were older than 3 years. We examined 191 German Shepherds, 65 Malinois, 14 huskies, 12 mongrels, ten terriers, four Rottweiler, three Labrador retriever, three Pekingese, two Lolo foxes, two Boo dogs, two Cocker spaniels, one sheepdog, and one Hawshar dog. All dogs were clinically examined, and body temperature, pulse, respiratory rate, and clinical signs of babesiosis were recorded.

### Sample collection

Blood samples were collected from the cephalic vein and were placed in 2-mL EDTA tubes for microscopic examination; after this, samples were frozen until DNA isolation and PCR.

### Molecular genetic assay

#### DNA isolation

A genomic DNA extraction kit was used to isolate DNA from blood (ReliaPrep^™^ Blood gDNA Miniprep System; Promega, USA). Quality and contraption of isolated DNA were assessed using a NanoDrop system (Thermo Scientific, Waltham, USA) and by agarose gel (1%) electrophoresis using RedSafe Nucleic Acid Staining Solution (iNtRON, Seongnam, Korea). DNA purity ratios of 1.6-1.9 at 260/280 nm were observed [19].

#### PCR protocol

We used primers targeting a 450-bp fragment of the 18S rRNA gene of *B. canis*, PIRO-A1 (5`-AGG GAG CCT GAG AGA CGG CTA CC-3`) and PIRO-B (5`-TTA AAT ACG AAT GCC CCC AAC-3`) [20,21]. The total PCR reaction volume of 25 μL included 12.5 μL 2× master mix containing 50 units/mL *Taq* DNA polymerase supplied in reaction buffer (pH 8.5), 3 mM MgCl_2_, 400 μM of each dNTP, 0.5 μL of each primer (final concentration 10 pmol), 2 μL template DNA, and 9.5 μL nuclease-free water. All reagents were obtained from Promega. We used a thermal cycling protocol as described previously [10], comprising initial denaturation at 94°C for 10 min, followed by 40 cycles of denaturation at 94°C for 30 s, annealing at 60°C for 30 s, extension at 72°C for 30 s, and a final extension step at 72°C for 5 min. PCR products were visualized using agarose gel (1.5%) electrophoresis (Bio Basic, Canada), and gels were photographed under UV radiation using a digital camera (Bio-Rad Laboratories, USA).

#### Sequencing and phylogenetic analyses

PCR products were commercially sequenced (Macrogen, Korea) using the forward primer, and the sequences were aligned and compared with reference sequences of the 18S rRNA gene of *B. canis* that was available in the NCBI database using BLAST (http://www.ncbi.nlm.nih.gov/BLAST/). Phylogenetic trees were produced, and sequences were analyzed by multiple sequence alignment analyses using Molecular Evolutionary Genetics Analysis software (MEGA 6.0).

### Statistical analysis

SPSS version 20.0 (IBM Corp., NY, USA) was used for statistical analysis. Chi-square tests were used for comparisons of prevalence levels (at p≤0.05), and multiple logistic regression analyses (odds ratio) were used to estimate potential babesiosis risk factors.

## Results

**Figure-1:**
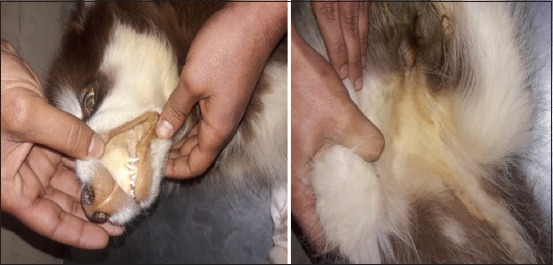
The dogs infected with *Babesia canis* suffered from severe blood hemolysis and icterus.

Clinical signs in dogs with *B. canis* infections varied from acute phase and chronic to the development of carrier statuses with slight or inconspicuous clinical manifestation. Clinical signs differed between 16 infected dogs (Table-1), and the main signs were malnutrition, anorexia, and tick infestation (in five dogs; 31%), vomiting and diarrhea (in three dogs; 18%), and pale mucus membranes (in eight dogs; 50%); in two dogs, sever icterus of the mucus membranes was observed after death (Figure-1). Clinical parameters included body temperatures of 37.7-40.5°C (only five dogs had a fever), respiratory rates of 19-69, and pulse rates of 60-136. Infected dogs showed no signs of urinary system such as oliguria, anuria, or hemoglobinuria. Ticks infestation was assessed as present or absent; however, ticks were not taxonomically identified.

**Table-1:** The clinical signs of *Babesia canis* spp. among 16 dogs.

Clinical signs	Dogs (%)
Malnutrition	5 (31)
Depression	9 (56)
Anorexia	8 (50)
Nervous manifestation	2 (12.5)
Ticks infestation	5 (31)
Vomiting and diarrhea	3 (18)
Pale mucus membrane	8 (50)
Icterus and dead	2 (12.5)
Heartbeat weak	7 (43.7)
Irregular heart rhythm	6 (37.5)

**Figure-2:**
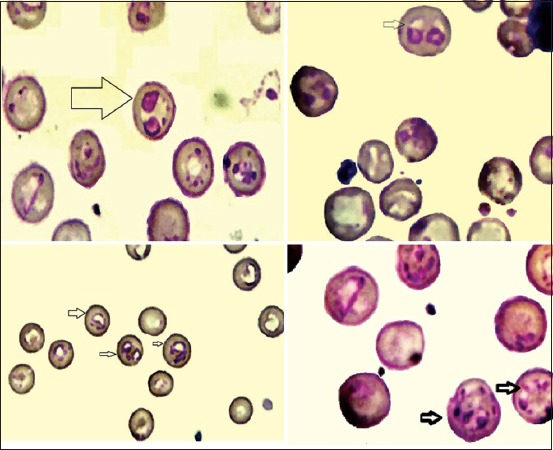
*Babesia canis* inside canine erythrocytes (intracellular) stained by Giemsa stain at (Light microscope [100×]).

**Figure-3:**
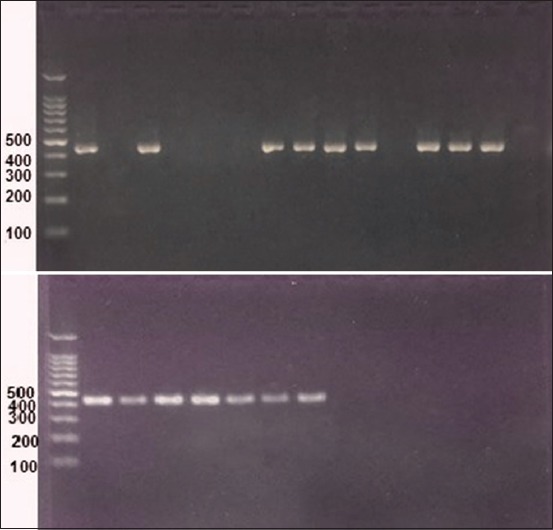
*Babesia canis* polymerase chain reaction amplicons of 18S rRNA gene, 16 positive cases at 450 bp piro A1 and piro B primers (red safe stain), the first lane 100 bp DNA ladder.

Microscopic diagnosis revealed paired or single pear-shaped intra-erythrocyte large piroplasm *B. canis* (Figure-2), and out of 310 blood smears, *B. canis* spp. was observed 5 times, *B. canis canis* 4 times, and *Babesia canis vogeli* once. In contrast, PCR amplification and sequencing produced 16 cases of *B. canis* spp. (Figure-3), 14 cases of *B. canis canis* (4.51%), one case of *B. canis vogeli* (0.32%), and one case of *B. canis rossi* (0.32%). The total prevalence of *B. canis* *spp*. infections in dogs was 5.16% as determined by PCR, which was substantially higher than infection prevalence determined by microscopy 1.61% (Table-2).

**Table-2:** Comparison between microscope and PCR for diagnosis *Babesia canis* spp.

Statues	Total dogs	Infected dogs**	Percentage
PCR	310	16*	5.16
Microscope	310	5	1.61

* Refer to increase significant at p≤0.05 (Chi-square:5.7; df: 1; p=0.01). PCR=Polymerase chain reaction

The sequences of 18S rRNA gene were made available at NCBI GenBank under the following accession numbers: *B. canis canis*: MN339533.1, MN339534.1, MN339535.1, MN339536.1, MN33 9537.1, MN339538.1, MN339539.1, MN339540.1, MN339541.1, MN339542.1, MN339543.1, MN 339544.1, MN339545.1, and MN339546.1; *B. canis rossi*: MN339547.1; and *B. canis vogeli*: MN 339549.1. Variation in taxonomic identification depended on query coverage, and out of 16 positive cases (5.16%), 14 were infected by *B. canis canis*, and *B. canis rossi* and *B. canis vogeli* had infected one dog each.

**Figure-4:**
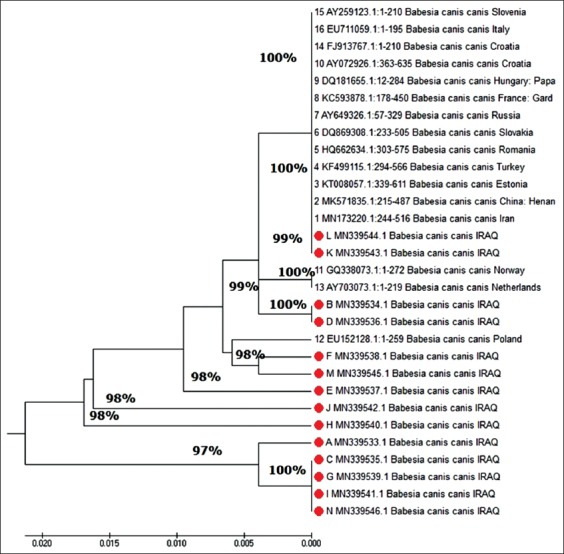
Phylogenetic tree of 18S rRNA gene of *Babesia canis* *canis*.

A phylogenetic tree (Figure-4) of *B. canis* showed that the 14 *B. canis canis* isolates observed in the present study clustered in three clades: Clade 1 (MN339543.1 and MN339544.1) with 99% similarity between isolates, clade 2 (MN339534.1 and MN339536.1) with 100% similarity, clade 3 (MN339535.1, MN339539.1, MN339541.1, and MN339546.1) with 100% similarity, and several isolated lineages(MN339533.1, MN339537.1, MN339538.1, MN339540.1, MN339542.1, and MN339545.1) with 97% - 98% similarity to other isolates. Phylogenetic analyses of *B. canis* isolates from different geographical regions (Slovenia, Croatia, Italy, Hungary, France, Russia, Romania, Turkey, Estonia, and Iran) showed 100% similarity with clade 1 of the present study, and 99% showed similarity with clade 2, and clade 3 showed 97% similarity with isolates from different countries, while the isolated lineages of *B. canis canis* isolates of the present study showed 97%-98% similarity to other isolates. *B. canis rossi* was 99% similar to an isolate from Turkey (MK918605.1) and 100% similar to an isolates from Montserrat, Spain (KP221649.1), and the USA (HM585429.1). *B. canis vogeli* identified in the current study was closest to the specimens from Japan (AB083374.1) and Sudan (DQ111756.1; 98% similarity, each). The frequency of substitutions in *B. canis* nucleotide sequences in the present study revealed that a total of 44 transition substitutions and 27 transversion substitutions were observed in the 16 isolates, and the predominant substitutions were A >G (25%) and T >C (26%).

Infection predisposition was examined by sex, and age (Table-3), and we found a higher but statistically non-significant risk of *B. canis* infection in males (odds ratio 1.81) compared to females. The probability of infection was higher in young dogs of up to 3 years of age than in older individuals (odds ratio 1.78), which was, however, not significant. *B. canis* infections were significantly more frequent in husky dogs (odds ratio 6.82) than in German Shepherds (Table-4). *B. canis* most commonly occurs in March, April, May, June, and July (Table-5), and the highest incidence was observed in April(13.7%) and June (10.2%). For statistical analyses, study months were assigned to three seasons, i.e., winter, spring, and summer, and the risk of *B. canis* infection in winter was zero; infection risk was higher in spring (odds ratio 1.73) than in summer; however, this difference was not significant.

**Table-3:** Predisposition of sex and age in 16 dogs infected by *Babesia canis spp*.

Variables	Infected/Total dogs
Sex	
Male	12/193 *
Female	4/117
Age (years)	
<3	11/171**
>3	5/139

* Confidence interval (CI 95%)=0.57-5.77, Odds ratio=1.81;

** Confidence interval (CI) 95%=0.60-5.26, Odds ratio=1.78

**Table-4:** Effect of breeds in 16 dogs infected by *Babesia canis* spp.

Breed	Infected/Total	Significant
German shepherd	7/191	B
Malinois	4/65	
Husky*	3/12	A
Crossbreed	2/14	
Other breed	0/28	

* Confidence interval (CI 95%)=1.56-29.75, Odds ratio=6.82.

**Table-5:** Number of infected dogs through months out of 16 dogs infected by *Babesia canis* spp.

Months	Infected/Total dogs	Infected/Total dogs
December-2018	0/25	Winter 0/75
January-2019	0/25	
February-2019	0/25	
March-2019	2/25	Spring* 12/149
April-2019	4/29	
May-2019	1/46	
June-2019	5/49	
July-2019	4/36	Summer
August-2019	0/25	
September-2019	0/25	4/86

* Confidence interval (CI 95%) = 0.54-5.53, Odds ratio=1.73.

## Discussion

We described important clinical signs of babesiosis, such as malnutrition, anorexia, depression, and paleness of mucus membranes; icterus typically only occurs in the final stage of the disease and is associated with imminent death (as observed in two out of 16 infected dogs). Icterus was a non-significant sign, and we observed icterus in 12.5% of infected animals; however, 50% of all infected dogs showed pale mucus membranes. A previous study reported icterus in only 7.5% of infected dogs [15], whereas Davitkov *et al*. [14] observed icterus in 28% of dogs with babesiosis. In the present study, pale mucus membranes were substantially more common than icteric mucus membranes during *B. canis* infection which is likely due to severe anemia, whereas icterus only occurs after severe hemolysis in the final stage of this disease. Thus, *B. canis* can lead to fatal hemolytic anemia, which is typically associated with icteric mucus membranes.

In the present study, malnutrition was associated with chronic cases and long periods of anorexia; these signs typically occurred together with anemia, which is in line with the previous observations [15,22]. Some infected dogs had a fever with body temperatures up to 40.5°C; however, this only occurred in the acute and subacute stages. Babesiosis is typically associated with febrile illness due to the production of endogenous pyrogens following hemolysis, immune responses to against parasite infection, and activation of inflammatory mediators during the acute and subacute phase [23]. In the current study, clinical signs associated with the acute and subacute phase included pale mucus membranes or icterus, tick infestation, febrile illness, and hemolysis, whereas malnutrition, anemia, and anorexia were considered symptoms of a chronic course of the disease.

According to sequencing data, *B. canis canis* was the most common species of *Babesia* in Baghdad, Iraq, with an infection prevalence of 4.51%, while the prevalence of *B. canis vogeli* was 0.32% and that of *B. canis rossi* was 0.32%. Total infection prevalence was 5.1%, as detected by PCR; in contrast, *B*. *canis* infections were detected in only five dogs using blood smears (1.61%), indicating a substantial number of false-negative results with this method. This is in line with the results of Da Silva *et al*. [24] who found low prevalence using microscopy diagnosis than with molecular methods. This considerable discrepancy in detection success is most likely due to the high sensitivity of PCR-based diagnosis and low parasitemia, particularly during the chronic phase or during carrier state.

Infection rates have been estimated based on molecular genetic methods by numerous researchers: *B. canis* infection prevalence was 3.3% in Sao Luis, Brazil [25], 3.75% in Iran [1], and 3% in India (*B*. *canis* *vogeli*) [26], all of which are below the prevalence observed in the current study; in Recife, Brazil, this value was 4.8% [14], which approximates that observed in the present study; however, other studies recorded the prevalence of 10.7% [27], 22.5% [15], 54% [8], suggesting differences between countries, effects of study duration, and differences in control measures. Moreover, the geographical distribution of ticks and climate conditions facilitating transmission of *B. canis* also likely affect local prevalence. Local and seasonal distribution and abundance of ticks are, therefore, important epidemiological indications [26,27].

Transmission pathways are also important to consider, as direct transmission through dog bites is very likely in some studies [14]. In the current study, ticks were observed on 31% of infected dogs, which were within the range of previously reported tick infestation during canine babesiosis (55-14.8%) [14,28]. However, no significant correlation of tick infestation and hemoparasites has been found [28]. While no taxonomic data on ticks were collected in the present study, a previous study found *R. sanguineus* in 21.56% of dogs in Baghdad Province [29].

Phylogenetic analyses of 18S rRNA sequences produced three clades, with clade 1 comprising two isolates obtained from German Shepherd dogs, which differed by one nucleotide transition (A to G). Clade 2 differed from clade 1 by 1-2 nucleotide substitutions, and infected dogs of this clade were present in the same region in Baghdad Province. Clade 3 showed 97% similarity with other clades or isolates and varied in five nucleotide positions. Homology analyses of one isolate to each *B. canis rossi* and *B. canis vogeli* revealed polymorphisms in two and four nucleotide positions, respectively, and *B. rossi* showed 99% similarity with isolates from other geographical regions. *B. canis rossi* occurred in a Malinois dog of 2 years of age in March, while *B. canis vogeli* was isolated from a German Shepherd of 6 years of age in July. *B. vogeli* showed 98% similarity with isolates from other geographical regions.

Our results suggest that sex may be a risk factor for *B. canis* infection, as infection prevalence was higher in males than in females, which is in line with the results of several previous studies [24,30,31]; ­however, female-biased *B. canis* infection prevalence has also been reported [1,23]. In the current study, dogs younger than 3 years showed higher infection prevalence than older individuals, which have been reported previously [1,15,24]. This may be due to their immature immune system [15]. In Iraq, the typical vaccination protocol comprises polyvalent vaccines against seven diseases, and vaccination against canine distemper and parvovirus are typically administered to young dogs. According to the previous study, it found that the stress factor due to vaccination has an effect on the immune system of young dogs, which is crucial for fighting off *B. canis* infections [3].

Our results indicated that huskies and mongrels were more susceptible to *B. canis* infection than Malinois and German Shepherd dogs; however, the previous studies found comparably high infection rates in German Shepherds [15,23,32]; however, in the present study, *B. canis* infection was significantly more common in huskies than in other breeds and was more common in mongrels than in other breeds, which was not significant. We, therefore, suggest low tolerance of huskies to the environment in Iraq, which makes them more susceptible to diseases; moreover, mongrels are typically stray dogs that are exposed to hard climate conditions, which may also contribute to reduced immunity.

The highest incidence of canine babesiosis was observed in April and June, which is similar to the results of Tayyub *et al*. [33]. Furthermore, the highest number of positive cases occurred in spring and summer, likely because of increased exposure to ticks during these seasons [1] and warm and humid climate conditions favoring disease transmission by vectors [33].

## Conclusion

The results of the present study indicate that *B. canis canis* is the predominant species causing *Babesia* infections in dogs in the region of Baghdad, Iraq. PCR assay and sequence analyses are more sensitive diagnostic methods than microscopy examination. Furthermore, potential risk factors such as sex, age, and period of infection were investigated in this study, and the results suggest that the frequent monitoring of canine babesiosis in Iraq would be required to reduce the prevalence of *Babesia*.

## Authors’ Contributions

NMB: Study idea, planning, and design. Conduct of the clinical, hematological, molecular methods, statistical analysis, and manuscript writing. AAY: Supervisor and guidance in the study, results interpretation, and revision of the manuscript. All authors read and approved the final manuscript.
